# Patient perspectives of lithium and quetiapine augmentation treatment in treatment-resistant depression: A qualitative assessment

**DOI:** 10.1177/02698811221089042

**Published:** 2022-04-27

**Authors:** Lucas McKeown, Rachael W Taylor, Elana Day, Rupal Shah, Lindsey Marwood, Helena Tee, Jess Kerr-Gaffney, Emanuella Oprea, John R Geddes, R Hamish McAllister-Williams, Allan H Young, Anthony J Cleare

**Affiliations:** 1 Centre for Affective Disorders, Department of Psychological Medicine, Institute of Psychiatry, Psychology & Neuroscience, King’s College London, London, UK; 2 Department of Psychiatry, Warneford Hospital, University of Oxford, Oxford, UK; 3 Warneford Hospital, Oxford Health NHS Foundation Trust, Oxford, UK; 4 Cumbria, Northumberland, Tyne and Wear NHS Foundation Trust, Newcastle upon Tyne, UK; 5 Northern Centre for Mood Disorders, Newcastle University Translational and Clinical Research Institute, Newcastle upon Tyne, UK; 6 South London and Maudsley NHS Foundation Trust, London, UK

**Keywords:** Treatment-resistant depression, pharmacotherapy, major depressive disorder, qualitative, thematic

## Abstract

**Background:**

Treatment-resistant depression (TRD) has a profound cost to patients and healthcare services worldwide. Pharmacological augmentation is one therapeutic option for TRD, with lithium and quetiapine currently recommended as first-line agents. Patient opinions about pharmacological augmentation may affect treatment outcomes, yet these have not been systematically explored.

**Aims:**

This study aimed to qualitatively assess patient experiences of lithium and quetiapine augmentation.

**Methods:**

Semi-structured interviews were conducted with 32 patients from the ongoing lithium versus quetiapine open-label trial comparing these augmentation agents in patients with TRD. Interviews were audio recorded, transcribed and a thematic analysis was used to assess patient opinions of each agent.

**Results:**

Four main themes were generated from the thematic analysis: ‘Initial concerns’, ‘Experience of side effects’, ‘Perception of treatment efficacy’ and ‘Positive perception of treatment monitoring’. Patient accounts indicated a predominantly positive experience of lithium and quetiapine augmentation. Greater apprehension about side effects was reported for lithium prior to treatment initiation, but greater experience of negative side effects was reported for quetiapine. Clinical monitoring was perceived positively.

**Conclusion:**

Patient accounts suggested treatment augmentation with lithium or quetiapine was acceptable and helpful for most patients. However, anticipation and experiences of adverse side effects may prevent some patients from benefitting from these treatments.

## Introduction

Up to 70% of patients with depression continue to experience impactful symptoms after receiving first-line recommended pharmacological treatments, such as selective serotonin reuptake inhibitors (SSRIs) and serotonin-norepinephrine reuptake inhibitors (SNRIs) (Ruberto et al., 2020). Treatment-resistant depression (TRD) is defined as suboptimal responses to two or more antidepressant trials of adequate dosage and duration in the current depressive episode (Fekadu et al., 2018), with rates that may exceed 40% of patients (Nierenberg et al., 2006). Patients with TRD have significantly higher rates of mortality, poorer prognosis and twice the individual healthcare costs of patients with non-refractory depression (Fekadu et al., 2009; Reutfors et al., 2018; Sussman et al., 2019). Many patients with depression are undertreated (Fernández et al., 2007), and adequate treatment of TRD can improve prognosis (Wooderson et al., 2014), highlighting the importance of appropriately treating the condition.

Therapeutic options for patients with TRD include increasing the dose of ongoing antidepressant treatment, switching to an alternative antidepressant, combining antidepressants or augmentation with another agent (Cleare et al., 2015). There is evidence that pharmacological augmentation is effective in TRD (Strawbridge et al., 2019), with lithium and quetiapine among the agents with the strongest support. Lithium is an enzyme modulator while quetiapine has multiple modes of action as a dopamine, serotonin and adrenergic antagonist. Both lithium and quetiapine are recommended by most major treatment guidelines (Taylor et al., 2020), but the existing evidence does not favour one over the other, leading to clinical equipoise (Bauer et al., 2013). However, despite evidence of efficacy and guideline recommendations, augmentation is underused in clinical practice and there remains a proportion of patients who do not find augmentation adequately helpful (Day et al., 2021).

Tolerability varies between augmenting agents (Strawbridge et al., 2019), and may be one contributing factor to suboptimal response, but qualitative assessment of patients’ perspectives may highlight other possible causes (Green and Britten, 1998). Qualitative research can describe and interpret complex phenomena involving the views, beliefs and experiences of patients (Pope et al., 2002) and identify subjective factors influencing patient outcomes (Rusinová et al., 2009). Despite evidence that patient opinions have a direct impact on treatment outcomes (Laferton et al., 2017; Mergl et al., 2011), qualitative accounts have rarely informed current treatment guidelines (McPherson and Beresford, 2019). To our knowledge, patient opinions concerning the adjunctive use of lithium and quetiapine in TRD have not been qualitatively assessed. Such research could help identify barriers to positive outcomes that have not been identified by quantitative research.

This study aimed to qualitatively assess opinions of lithium and quetiapine augmentation in patients with TRD, in order to identify factors contributing to suboptimal outcomes and novel indications for the recommendation of one agent over the other. All participants were taking part in the lithium versus quetiapine in depression (LQD) study, an ongoing open-label randomised controlled trial (RCT) investigating the clinical and cost effectiveness of lithium and quetiapine augmentation in TRD (Marwood et al., 2017). This study was an optional add-on qualitative study embedded within the larger LQD trial.

## Methods

Ethical approval was obtained from NHS East of England – Cambridge South Research Ethics Committee (reference 16/EE/0318).

### Participants

LQD study participants were invited to take part in this optional add-on study at their final research assessment, 52 weeks post-randomisation to lithium or quetiapine, between November 2018 and February 2020. Briefly, the LQD study recruited adult patients with current depression and ⩾2 antidepressant treatment failures in their current depressive episode. Patients were randomised 1:1 to open-label lithium or quetiapine, administered in addition to their existing antidepressant treatment. Patients were followed up for 12 months and completed research assessments at baseline, 8, 26 and 52 weeks, in addition to weekly online self-report measures. The LQD study protocol has been published in full (Marwood et al., 2017). Willing LQD participants provided written informed consent and completed semi-structured interviews with a member of the LQD research team for this study.

### Interview procedures

Interviews were conducted by a researcher and guided by an interview schedule (see Supplementary Material). Researchers were mostly female and held a relevant undergraduate or master’s degree. L.M. and R.W.T conducted training with all researchers. Typically, participants had a pre-existing relationship with the researchers as they had previously conducted their LQD study assessments. Interviews were audio recorded and transcribed verbatim. Transcription was completed utilising a naturalised approach meaning that speech was expressed in writing as it was said, without being filtered by transcribers (Oliver et al., 2005). Transcripts were later analysed using NVivo software (QSR International Ltd., 2012).

### Data analysis

Data was analysed using thematic analysis, a widely used approach to identify patterns and higher levels of meaning within qualitative data (Braun and Clarke, 2006). Thematic analysis was chosen as it is considered appropriate for analysing the views and experiences of patients (Marks and Yardley, 2004). A data-driven, inductive approach was adopted, following the six stages outlined by Braun and Clarke (2006). The first stage of analysis involved reading and rereading each transcript several times while noting information of interest to the research question. In phase two, the data were coded by systematically identifying and highlighting features of interest and labelling them in accordance with their content. In the third phase, codes were clustered together to form any larger overarching themes. The fourth phase involved the refining of themes which led to the removal of certain themes, and the collating of others. For this stage of the analysis, two additional researchers (E.D. and R.S.) conducted an independent review of the themes to ensure coded extracts were accurately reflective of each theme and that themes were reflective of the data set. Finally, themes were named as appropriate, and any discrepancies were resolved by consensus.

## Results

Thirty-two participants (16 randomised to each treatment arm) agreed to take part in the study (see Table 1 for demographic and clinical characteristics). Four main themes and 11 subthemes were identified in the thematic analysis: ‘Initial concerns’, ‘Experience of side effects’, ‘Perception of treatment efficacy’ and ‘Positive perception of treatment monitoring’.

**Table 1.** Participant demographic and clinical characteristics (*n* = 32).

Age (years), mean (SD)	43.1 (15.0)
Gender, *n* (%)	
Female	19 (59.4)
Male	13 (40.6)
Employment status, *n* (%)	
Employed	20 (62.5)
Unemployed	9 (28.1)
Retired	2 (6.3)
Student	1 (3.1)
Education level, *n* (%)	
Primary education or less	2 (6.3)
Secondary education	8 (25.0)
College-level education or equivalent	8 (25.0)
Degree-level education/diploma	11 (34.4)
Postgraduate degree	3 (9.4)
HDRS-17 score, mean (SD)	21.3 (5.1)
Number of past episodes of depression, mean (SD)^a^	2.4 (2.2)
Number of antidepressant treatment failures in current episode, *n* (%)	
2	10 (31.3)
3	10 (31.3)
4	7 (21.9)
5+	5 (15.6)
Number of axis 1 comorbidities^b^, *n* (%)	
0	9 (28.1)
1	8 (25.0)
2	5 (15.6)
3+	10 (31.3)
NHS trust, *n* (%)	
South London and Maudsley NHS Foundation Trust	15 (46.9)
Cumbria, Northumberland, Tyne and Wear NHS Foundation Trust	8 (25.0)
Oxford Health NHS Foundation Trust	9 (28.1)

HDRS-17: Hamilton Rating Scale for Depression, 17 item version; NHS: National Health Service; SD: standard deviation.

a *n* = 29 due to missing data.

b DSM-5 diagnoses assessed using the Mini-International Neuropsychiatric Interview.

### Theme 1: initial concerns

Participant narratives revealed a number of concerns about both medications before treatment began. Typically, discussion around concerns was in response to the first question, which explored participant opinions before randomisation. Concerns were characterised by two interrelated subthemes ‘Apprehension towards lithium’ and ‘Worry about side effects’.

#### Apprehension towards lithium

Prior to initiation, nine participants (six of whom had been subsequently randomised to quetiapine and three to lithium) held beliefs that lithium might be a more ‘extreme’ treatment than quetiapine. Reasons for expressed apprehension differed. For some, awareness of the necessity for blood tests to ensure lithium levels remained in the therapeutic range and the possibility of lithium toxicity resulted in greater apprehension at the prospect of taking lithium compared to quetiapine:

P: It seemed the more extreme of the two options, erm . . . was hoping that I wouldn’t be randomised to that one but I was . . . just in terms of like the blood tests and the monitoring . . . (P23)

Other concerns were generated from reading the information sheet about lithium prior to treatment. Some described feeling worried about specific side effects listed:

P: Reading about the two, the quetiapine seemed like the less scary option. (P23)

P: I read about them and I um I thought that um lithium seemed like it would be harder on the digestive system than quetiapine might. (P14)

Some felt apprehensive following their own research prior to the study:

P: I think like stuff that you read it isn’t really science based, I think it’s a bit scary if you don’t . . . until you understand actually it’s just another, another treatment. (P23)

Others simply held a negative preconception of lithium based on their feelings towards it before randomisation:

P: I had quite a negative feeling towards it – I don’t know maybe in my subconscious I felt it wasn’t didn’t feel – like something I wanted to take. (P19)

#### Worry about side effects

One-quarter of participants stressed concerns regarding the potential side effects of lithium and quetiapine treatment before the trial. For example, several felt concerned that sedation might compromise their daily life functioning and performance at work:

P: I was worrying if I was just going to be falling asleep at work. (P8)

Others were concerned at the potential for weight gain, which was listed as an adverse effect for both agents:

P: the main thing about whether or not I want to take part, and I went back for ages, was worrying about like the weight gain. (P2)

P: coz like I had an eating disorder like the weight thing was quite a big issue for me . . . I was just really really worried about that. (P2)

For some, the need to effectively treat their depression seemingly outweighed these concerns:

P: weight gain was obviously a concern, but I had to weigh up my mental health and, you know, a bit of weight gain. So you know, that was it. (P7)

P: I think I’ve got to the point where I’d tried 8 antidepressants and still felt awful so I was like, I’ll give it a go (laughs) it can’t – it can’t be worse than the side effects of anything else. (P16)

### Theme 2: experience of side effects

Twenty-one accounts described encountering side effects from their augmentation treatment. Most felt that they were manageable, and continued treatment, but some felt that the side effects resulted in a negative treatment experience. Participants randomised to lithium augmentation generally described their side effects as milder than those receiving quetiapine.

#### Side effects with quetiapine

Side effects with quetiapine were near universal. Generally, patient narratives indicated feelings of sedation and unwanted weight gain to be most troublesome.

For some, feelings of sedation were overbearing and were thought to negatively impact their treatment outcome:

P: side effects were just exhaustion and tired um almost comatose in the mornings. (P6)

P: it was horrible I would sort of wake up in the morning and still be like I hadn’t had a-a night’s sleep. (P6)

P: I was just completely wiped out all the time, I mean I was just zonked permanently basically so it wasn’t a feasible option to go forward basically. (P22)

However, two respondents felt sedation helped to improve their sleep which was previously disrupted:

P: it definitely helps so much with my sleep erm, because my sleep used to be really bad . . . whereas now like I know I take it and then like within half an hour I fall asleep. (P2)

Issues with unwanted weight gain and appetite increase were also among the most commonly reported side effects for quetiapine:

P: having a plan for um . . . for weight management is a good, is a good thing to do um . . . cuz that’s been, that’s been pretty difficult. (P14)

P: one thing that’s caught me out is the appetite that I just get big bursts of cravings of food. (P8)

The number of side effects reported differed, and for some, several different side effects were reported. Alongside sedation and weight gain, one participant perceived quetiapine to be worsening other pre-existing symptoms:

P: sleep and weight gain and I also like my um . . . the level of uh constipation and diarrhoea and how much that’s affect, been affecting me . . . it certainly hasn’t improved um it might have gotten worse since I started taking quetiapine. (P14)

#### Side effects with lithium

Patients receiving lithium generally indicated side effects to be more manageable. Despite this, several participants did report negative experiences.

Some described issues with tremor. Mostly, this appeared manageable and unproblematic:

P: Just a little bit of a shudder in the hands when I was trying to do something gently but that’s neither here nor there. (P27)

P: only issue that I’ve had that I think is actually related to the medication is err tremor that comes and goes but it’s mild and it doesn’t cause me any issues. (P18)

However, one subject described their tremor as severe, leading them to contact the study team for support:

P: Side effects were shakes, shakes you know, until I got shakes . . . I was trying to text you I think and then . . . Bad it was. (P3)

Several patients discussed issues relating to dry mouth. Despite being aware of the potential for dry mouth before treatment, one participant emphasised the disparity between their perception and the reality of experiencing it:

P: it’s one thing to hear someone say it and then to live through it um . . . it just literally is just a constant sort of dry mouth and not quite a constant thirst but you really have to discipline yourself to just keep drinking. (P13)

Vivid dreams were not listed as an adverse effect for lithium but were discussed by one participant:

P: The bad things were the dreams. They were basically making it a night reality . . . and certain dreams that you have can be quite frightening. (P26)

One participant discussed adverse effects on the kidney resulting in treatment discontinuation:

P: for me it would be the kidney side effects, you know. That’s what damaged me. (P25)

#### Managing side effects

Several subjects volunteered information about the action they took to better manage side effects.

Participants talked about the importance of the time they took their quetiapine medication each day:

P: we started taking it earlier and then it would help me sleep and it sort of got to manage that those side effects. (P6)

P: and also like play with what time of the evening is right to take the medication before you go to bed um . . . because that uh was definitely a balance to strike. (P14)

Several participants taking lithium described efforts to increase water consumption. One recognised that increasing their water intake could reduce the severity of their side effects:

P: I felt like some of the side effects got better because I was actually like staying hydrated and things. (P16)

For another, managing their weight gain enabled them to make positive changes:

P: being on the . . . quetiapine helps me, like I haven’t been – stepped inside a gym or swimming pool for like over seven years so, for me to go swimming and stuff is a – is a big thing so, it’s helping me um do that. (P7)

### Theme 3: perception of treatment efficacy

Another primary topic of participant discussion surrounded the perceived efficacy of their medication. Participants in this sample reported their experience of both medications as largely positive, with over half of the sample perceiving benefits. However, not all perceived benefits appeared to be of the same magnitude. Some felt their treatment had ‘worked’ whereas others felt their symptoms had become more manageable upon treatment. Unfortunately, over one-quarter of participants did not report feeling any benefits from their medication.

#### Improvement in symptoms

Six participants taking lithium described considerable improvements in their symptoms and mood:

P: I’ve come from down here, say naught, up to ninety-nine honestly, the amount of people that have said how different I am in this last year, and I’ve been down for years so this is great for me absolutely great. (P4)

Six participants taking quetiapine also reported large improvements, with some specifically mentioning changes in anxiety and panic symptoms:

P: the most major thing was not having all the anxiety and panic attacks because that strips away all the really good emotions that you feel . . . So um it’s been nothing but a godsend really. (P7)

Others described the key benefits of their treatment as better illness management and greater ability to cope within difficult circumstances:

P: there were times of the year as well as situations that usually would uhh would trigger depressive thoughts or sometimes even a depressive episode . . . I did have those difficult thoughts and you know a day where it was, when I was a bit down but it didn’t feel like depression, it didn’t feel like, it didn’t feel like hopelessness. (P14)

P: I would like to share the analogy of um . . . driving and being lost and it’s pointless carrying on driving and getting more lost if you can stop, get out the car and look at a map. Which is what the medication has helped me to do. (P8)

#### Mild and steady improvements

Some participants reported experiencing milder improvements in their symptoms.

One subject taking lithium reported this type of symptom change:

P: it was maybe two days out of the week that I felt a little better and five days I was still really, really low. From my point of view, I was going from zero positive days and so it was a benefit, it was a positive. (P22)

Others had difficulty delineating just how effective lithium was:

P: it’s kind of hard to tell, it’s not – I’m not exactly where I’d want to be um, but it’s . . . ok – it’s better than being where I was, so. (P17)

Some discussed the pace of improvement to be noticeably slow for lithium:

P: I can feel that there is something happening, but at a very slow pace though, but it doesn’t happen overnight. (P21)

Two patients did not perceive large improvements from taking quetiapine, but still felt it had been useful for them:

P: I think there was definitely a mild improvement. (P19)

P: no its uh – it’s been fine, it’s uh been worth trying. (P31)

#### Ineffective treatment(s)

Despite largely positive views of these medications, over one-quarter of participants did not perceive any benefits from treatment.

For some, the only noticeable change from taking quetiapine was the side effects:

P: I don’t notice a lot of change in myself um and that has been the case with all of my medications umm with the exception of the tiredness that is the only physical change that I have felt. (P6)

One participant did not perceive any improvement with dosages that corresponded with a safe lithium plasma level:

P: I just didn’t feel a . . . that much of a beneficial effect er until I was on a higher dose but then my blood levels showed that my lithium levels were too high so it had to go back down again. (P32)

Some participants reported self-blame for a lack of perceived symptom change. One participant treated with both medications described frustration at their failure to benefit from either one:

P: the fact that neither of them really worked . . . didn’t really dishearten me. I mean it . . . it’s frustrating from my personal point of view erm, that I feel like I’m rubbish basically you know it’s . . . it’s me. (P22)

Another who did not perceive benefits from lithium felt this could be due to their own expectations:

P: I think my personal problem was that I expected too much. (P29)

Some attributed failure to benefit as a consequence of difficult personal circumstances:

P: I’ve had such a difficult 12 months it’s then unfair in respect to the trial it’s been two deaths, a cancer scare and then 9 months off work through mental so it would be nice to have it on 12 months of normal. (P6)

### Theme 4: positive perception of treatment monitoring

Overall, participants reported the monitoring they received to be largely positive, with responses divided into three subthemes.

#### A good number of appointments

Participants were generally positive about the number of clinical appointments they were required to attend:

P: I felt like I I . . . it was useful to have that many. (P14)

P: the frequency, if anything gave me more positivity. (P22)

P: the frequent appointments, although I had to take time off work to come to them, they were really useful. Um I felt like I was being looked after, um and that I wasn’t really on my own in it, um which I think is something I kind of felt for a long time. (P17)

However, some felt the number of appointments might prove difficult:

P: it took an amount of effort, so I guess yeah I guess a good way to put it is if someone is particularly, suffering particularly badly . . . with depression, that might be an extra challenge. (P13)

#### Reassurance from quick communication

There was a sense of reassurance among participants regarding the speed and ease of communication with both the study team and clinicians.

For some, this was particularly useful when side effects became problematic:

P: I got a bit stressed about weight and stuff so I wanted to go down to a lower dose and it was like good, they were really quick to like reply and like get me an appointment with the psychiatrist . . . so that was really useful. (P2)

P: it’s been amazing um. So (doctors name) sees me regularly um and I can email if there’s any concerns as well, which s/he replies within 24 hours. (P7)

P: it was good cos I could contact um the researcher or the psychiatrist and get if there’s any worries I could contact anybody and they would get back to me quickly. (P15)

#### ‘Wasn’t as bad as I thought’ – blood tests were unproblematic

This subtheme refers specifically to patients treated with lithium which required clinical blood monitoring throughout treatment. Overall, patients showed a largely positive response to the required blood tests.

Some felt the blood tests were easier than they had initially perceived them to be:

P: it feels like quite a big undertaking with like the monitoring and the blood tests and stuff but it was fine, like it was easier than I . . . yeah it wasn’t a big, wasn’t a big problem at all. (P23)

P: I was first like startled by the amount of blood tests that I had to have, but I kind of got used to it so, apart from that it was absolutely fine. (P17)

Others described acceptance of the need for blood tests as a part of lithium treatment:

P: although you know, blood tests aren’t fun, it was good to have them because . . . you know it was, I needed to know what level it was in my blood um but yeah, overall I was happy with it. (P17)

P: They were definitely useful um obviously when there were more of them earlier on I had to sort of plan around them but I understand why it had to be like that. (P18)

## Discussion

This study aimed to explore patient perspectives of lithium and quetiapine augmentation for TRD. It important to note that this was a qualitative study embedded within the larger LQD study, and not designed for quantitative comparisons between treatments. Overall, however, patient accounts revealed a predominantly positive experience of augmentation, with over half perceiving benefits. Consistent with quantitative findings from previous studies, the participant accounts within this study highlighted several beneficial effects of both lithium and quetiapine, including reduction in symptoms and improvements in mood (Bauer et al., 2013; Undurraga et al., 2019). The magnitude of reported improvements varied, but even milder degrees of perceived change were described positively and were associated with better illness management and reductions in time spent feeling depressed. We found benefits were acknowledged both among participants treated with lithium and those treated with quetiapine, in line with existing quantitative evidence indicating parity of efficacy between lithium and quetiapine augmentation (Bauer et al., 2013).

Despite a predominantly positive perception, a significant minority of participants did not perceive benefits from treatment, and for some, this was attributed to their experience of adverse effects. However, we also found that prior to initiation, many participants experienced a conflict between their desire to effectively treat their depression, and their concerns about experiencing adverse effects. For some, learning about the side-effect profiles of these medicines before treatment made them debate their participation in the trial. This finding is consistent with previous research indicating that patients with TRD are both thoughtful and cautious about trying novel therapies (Lawrence et al., 2018). This highlights the importance of addressing concerns around adverse effects prior to initiation, which may contribute to the underuse of pharmacological augmentation in clinical practice. This may be particularly true for lithium, for which greater apprehension was reported prior to randomisation and treatment initiation.

Aligning with prior research, we found that experiences of adverse effects were nearly universal (Anderson et al., 2009; van Marwijk et al., 1990). Importantly, most patients could continue their treatment despite these adverse effects. Several patients assessed both the risks and benefits of their prescribed medication and felt the perceived benefits outweighed the disadvantages of the side effects they encountered. However, for a small portion of participants, adverse effects were overly impairing and, in some instances, this led to early withdrawal. Contrasting with prior studies comparing these drugs, we found that adverse effects were perceived as more challenging with quetiapine than with lithium (Dorée et al., 2007). Patients described difficulties with appetite increase, weight gain, restlessness and constipation, consistent with previously reported side effects associated with quetiapine (Bauer et al., 2009; El-Khalili et al., 2010). However, the most extensively discussed side effect with quetiapine was sedation. This aligns with findings from Bauer et al., (2013), who reported sedation to be the lead cause of discontinuation when comparing the tolerability of lithium and quetiapine in patients with TRD. However, our results also suggest that experiences of sedation may not always be negative; two participants reported positive experiences of sedation due to improvements in sleep. Our findings therefore underline the importance of considering individual patient characteristics and symptom experiences when making treatment decisions (Murphy and Peterson, 2015).

To a lesser extent, disruptive side effects were also reported with lithium. Consistent with quantitative evidence, participants experienced difficulties with dry mouth, tremor and nausea (Austin et al., 1991; Hawley et al., 1994). Side effects generally appeared manageable, although for two participants, difficulties with tremor and kidney function were thought to negatively impact their treatment outcome. Our findings could contribute to the development of patient information on side-effect profiles for use in clinical practice or future drug trials. Indeed, previous research has demonstrated the additional value of patient reporting of adverse effects for pharmacovigilance alongside healthcare providers’ reports (Avery et al., 2011).

Taken together, our findings indicate that there were a few differences in the perceived efficacy of lithium versus quetiapine. Greater apprehension about side effects was reported for lithium prior to treatment initiation, but greater experience of negative side effects was reported for quetiapine. This suggests that patient opinions prior to treatment do not necessarily align with treatment experience, and that anticipation of negative side effects may contribute to lack of uptake, especially for lithium.

Another key theme was the positive perception of clinical monitoring during treatment. Positive opinions of monitoring were near universal, even among those who did not perceive benefits from their medication. Participants felt there was plenty of opportunity to discuss concerns and felt reassured at how quickly they could achieve a response from clinicians and the study team. The number and frequency of clinical appointments were felt to be useful, and some participants felt this enhanced their treatment. Positive patient perceptions of monitoring complement the apparent clinical benefits of more active ‘measurement-based care’ approaches (Zhu et al., 2021).

Monitoring within the LQD study was guided by the Maudsley Prescribing Guidelines; lithium requires a higher degree than many other recommended pharmacological augmenters due to the need for frequent blood tests to establish a safe, therapeutic plasma level. Patients generally displayed acceptance and understanding of the need for blood tests; describing how they could ‘adapt’ to them over time. It has previously been suggested that clinicians are reluctant to prescribe lithium to patients with TRD because of the necessity for regular blood monitoring (Jollant, 2015). Given that regular blood tests were not perceived negatively in this study, our findings do not indicate that the need for additional monitoring should deter clinicians from prescribing lithium.

### Limitations

Participants were recruited at their week 52 LQD study assessment, meaning their discussion about pre-treatment and early treatment experiences required them to recall how they felt approximately a year prior, and therefore, recall accuracy may have impacted our results. Retrieval of autobiographical information is impaired in patients with depression, with less detailed and over-generalised recall predicting poor prognosis (Hitchcock et al., 2019; Sumner et al., 2010). Those who were still experiencing significant depressive symptoms at the end of the study may therefore have had poorer recall of their earlier thoughts and experiences of treatment. Relatedly, several LQD participants withdrew from the study before their week 52 assessments, or did not attend the assessment, and so could not be included. Although the factors contributing to withdrawal and non-attendance may vary, it is possible that this resulted in a bias whereby participants who had more negative experiences were not included in the present add-on study. Another limitation is the relatively small sample size in each group.

Finally, although the LQD study was a pragmatic study designed to reflect real-world practice, patients who are prescribed these medicines outside of a research context may not receive the same level of monitoring (Nikolova et al., 2018). During the LQD study, trial clinicians provided clinical monitoring and participants also had regular research assessments. It is possible that some participants did not differentiate the clinical appointments from the additional research ones when discussing their monitoring in this study, meaning their reported experience is not representative of general outpatient clinical practice. However, given the overwhelmingly positive response to the monitoring reported here, the experience of consistent monitoring appears to be an important part of treatment for patients and one that we suggest should be prioritised in clinical practice.

## Conclusion

Taken together, the patient narratives in our sample are supportive of lithium and quetiapine augmentation for the treatment of TRD. Although participants had initial concerns about taking these medications (particularly in the case of lithium), the majority found both lithium and quetiapine helpful in reducing their symptoms of depression. Side effects were common, yet most participants felt the benefits of treatment outweighed the disadvantages of the side effects they experienced. Our results must be interpreted in the context of a clinical trial; the regular monitoring received may not be representative of general outpatient clinical practice. Despite the often positive experiences described here, both medicines continue to be underutilised in clinical practice. Future qualitative work exploring clinicians’ perspectives on treatment augmentation may also be useful in identifying barriers to treatment and improving outcomes for individuals with TRD.

## Supplemental Material

sj-docx-1-jop-10.1177_02698811221089042 – Supplemental material for Patient perspectives of lithium and quetiapine augmentation treatment in treatment-resistant depression: A qualitative assessment Click here for additional data file. Supplemental material, sj-docx-1-jop-10.1177_02698811221089042 for Patient perspectives of lithium and quetiapine augmentation treatment in treatment-resistant depression: A qualitative assessment by Lucas McKeown, Rachael W Taylor, Elana Day, Rupal Shah, Lindsey Marwood, Helena Tee, Jess Kerr-Gaffney, Emanuella Oprea, John R Geddes, R Hamish McAllister-Williams, Allan H Young and Anthony J Cleare in Journal of Psychopharmacology
