# Supplementary material for: Patient perspectives of lithium and quetiapine augmentation treatment in treatment-resistant depression: A qualitative assessment
Source: J Psychopharmacol. 2022 Apr 27;36(5):557–65. doi: 10.1177/02698811221089042 (PMC9112618; doi:10.1177/02698811221089042)
Supplement: sj-docx-1-jop-10.1177_02698811221089042 – Supplemental material for Patient perspectives of lithium and quetiapine augmentation treatment in treatment-resistant depression: A qualitative assessment [file sj-docx-1-jop-10.1177_02698811221089042.docx]

# Qualitative Interview about views and experiences of LQD study medications

**QUESTION 1) Had you heard about lithium or quetiapine before taking part in the study?**

Prompt questions: If yes, what did you know about them? What did you think about them?

Did you know they were used to treat people with depression that had not fully responded to antidepressants? If no, quetiapine is an atypical antipsychotic medication, had you heard about those types of medication?

**QUESTION 2) What were your initial thoughts about lithium?**

Prompt questions: How did you feel about personally taking lithium? What were your expectations? What did you think it would be like? Did you think lithium would help? Were you concerned about any aspect of taking lithium?

**QUESTION 3) What were your initial thoughts about quetiapine?**

Prompt questions: How did you feel about personally taking quetiapine? What were your expectations? What did you think it would be like? Did you think quetiapine would help? Were you concerned about any aspect of taking quetiapine?

**QUESTION 4) Did you have a preference for either lithium or quetiapine before your treatment was allocated?**

Prompt questions: Was there one that you thought you’d prefer to take? If so, why? If not, why?

**QUESTION 5) Overall, how do you feel about the treatment that you were allocated to?**

Prompt questions: What were the good things about the medication? What were the bad things? Do you think that was the right treatment for you to take at that time? Is there another treatment you think would have been better?

**QUESTION 6) What do you think about or know about the treatment you were not allocated to [lithium/quetiapine]?**

Prompt questions: Do you think the other treatment would have been better or worse?

**QUESTION 7) How do you feel about the information you were given about the treatment before you started taking it? This could be the information or medication leaflets you received from the study team, information from your doctor or a pharmacist, or information your found online, for example.**

Prompt questions: Do you think you had enough information before starting the medication? What was the most useful source of information? What would have been useful to know or expect? From the information provided to you before taking the medication, what was useful to know?

**QUESTION 8) If you took or are taking the medication, what did you think about the number of appointments you had with doctors and the monitoring you had on the medication? For example, monitoring to see how well it was working, whether there were side effects, blood tests or physical checks.**

Prompt questions: Was it enough? Too much? Were these visits useful or problematic or useful in any way?

***Note to researcher: If required, clarify with participant that this does not include study visits with the research team***

**QUESTION 9) Is there any additional advice or information that you think others might find helpful for other people considering taking lithium or quetiapine?**

Prompt question: I there anything that you wish you had known about? Do you have any specific advice or pointers to share?

**QUESTION 10) Is there anything else that you would like to share about your experience of the medication?**
